# Ultra-Early Cranioplasty versus Conventional Cranioplasty: A Retrospective Cohort Study at an Academic Level 1 Trauma Center

**DOI:** 10.1089/neur.2022.0026

**Published:** 2022-08-01

**Authors:** Akal Sethi, Keanu Chee, Alia Kaakani, Kathryn Beauchamp, Jennifer Kang

**Affiliations:** ^1^ School of Medicine, University of Colorado, Aurora, Colorado, USA.; ^2^ Department of Neurosurgery, University of Colorado, Aurora, Colorado, USA.; ^3^ Division of Neurosurgery, Denver Health Medical Center, Denver, Colorado, USA.

**Keywords:** cranioplasty, decompressive craniectomy, traumatic brain injury, ultra-early cranioplasty

## Abstract

The goal of this study was to ascertain the efficacy, safety, and comparability of ultra-early cranioplasty (CP; defined here as <30 days from the original craniectomy) to conventional cranioplasty (defined here as >30 days from the original craniectomy). A retrospective review of CPs performed at our institution between January 2016 and July 2020 was performed. Craniectomies initially performed at other institutions were excluded. Seventy-seven CPs were included in our study. Ultra-early CP was defined as CP performed within 30 days of craniectomy whereas conventional CP occurred after 30 days. Post-operative wound infection rates, rate of return to the operating room (OR) with or without bone flap removal, operative length, and rate of post-CP hydrocephalus were compared between the two groups. Thirty-nine and 38 patients were included in the ultra-early and conventional CP groups, respectively. The average number of days to CP in the ultra-early group was 17.70 ± 7.75 days compared to 95.70 ± 65.60 days in the conventional group. The mean Glasgow Coma Scale upon arrival to the emergency room was 7.28 ± 3.90 and 6.92 ± 4.14 for the ultra-early and conventional groups, respectively. The operative time was shorter in the ultra-early cohort than that in the conventional cohort (ultra-early, 2.40 ± 0.71 h; conventional, 3.00 ± 1.63 h; *p* = 0.0336). The incidence of post-CP hydrocephalus was also lower in the ultra-early cohort (ultra-early, 10.3%; conventional, 31.6%; *p* = 0.026). No statistically significant differences were observed regarding post-operative infection, return to the OR, or bone flap removal. Our study shows that ultra-early CP can significantly reduce the rate of post-CP hydrocephalus, as well as operative time in comparison to conventional CP. However, the timing of CP post-DC should remain a patient-centered consideration.

## Introduction

Decompressive craniectomy (DC) is a common neurosurgical procedure that requires removal of part of the skull for management of intracranial hypertension after traumatic brain injury (TBI).^1–3^ Cranioplasty (CP) is routinely performed post-DC to restore the normal skull architecture, which can help maintain cerebral protection and cosmesis, as well as normalize cerebrospinal fluid and blood flow dynamics to promote neurological recovery and rehabilitation.^4–7^ Traditionally, CP has been delayed for as long as 3–6 months after the initial DC to allow for resolution of cerebral edema; however, timing has often been tailored to specific patient circumstances.^5,8^ Despite that CP is a common procedure, it still carries high rates of complications, ranging from 10% to 50%.^9,10^ Such complications can include infection, hydrocephalus, intracranial hemorrhage, cerebral edema, extra-axial fluid collection, bone flap resorption, and worsening of neurological deficits.^2,10^

Although post-operative complications are likely of multi-factorial etiology, extensive consideration has been given to the timing of CP and its effect on post-operative outcomes. Currently, there is no consensus on the optimal timing for performing a CP. Many studies have shown that early CP (1–3 months) is associated with a similar or lowered risk of post-operative complications compared to late CP (>3 months) and, in fact, may facilitate neurological recovery.^1,2,5,6,11–22^ Conversely, several studies have maintained that late, or delayed, CP has lower rates of post-operative complications,^23–28^ whereas others have found operative timing to be unrelated to post-operative complications.^29–31^ Few studies have assessed the effect of ultra-early CP (<30 days) on rates of post-operative complications in patients receiving DC.^9,16^ Therefore, the goal of this study was to ascertain the efficacy, safety, and comparability of ultra-early CP (defined as <30 days from original craniectomy) to conventional CP (defined as >30 days from original craniectomy) in patients receiving DC at a level 1 trauma center.

## Methods

### Study setting and patient eligibility

This is a single-center, retrospective cohort study gathering information on patients ≥15 years of age who have undergone DC with subsequent CP at the Denver Health Medical Center (Denver, CO). Denver Health Medical Center is a public, academic level 1 trauma center. All CPs performed were queried from January 2016 to July 2020.

All patients who had a craniectomy with a subsequent CP performed at Denver Health Medical Center were included in our analysis. After DC, all bone flaps were stored appropriately in dedicated human bone bank freezers. Exclusion criteria were as follows: 1) patients <15 years old; 2) patients who did not have their original craniectomy performed at our institution; 3) pregnant females; and 4) persons admitted from a correctional facility. Also, patients who had insufficient documentation in their electronic medical records were excluded from this study.

### Timing of cranioplasty

Patients were retrospectively grouped into either the ultra-early cohort (CP performed <30 days post-DC) or the conventional cohort (CP performed >30 days post-DC). The decision on whether to perform the CP either <30 or >30 days at our institution was dictated by the patients' clinical status. Primary factors incorporated into the clinical decision making of our neurosurgeons included improvement in Glasgow Coma Scale (GCS) score at the time of admission to the day of the proposed CP, improvement in functional neurological status, and, importantly, reduction in cerebral edema, particularly at the site of the DC.

### Data collection

Data were collected using the EPIC Systems electronic medical records from the Department of Neurosurgery at Denver Health Medical Center. Two distinct reviewers (A.S. and J.K.) queried patients' medical records from January 2016 to July 2020. Indications for DC included TBI, subdural hematoma, epidural hematoma, intracerebral hemorrhage, brainstem herniation, skull fracture, hydrocephalus, and ischemic stroke. Relevant data that were collected included patients' demographics, GCS at initial emergency room (ER) presentation, time span from DC to CP, operative length, occurrence of surgery-related infection, need for reoperation with or without the need for bone flap removal, and occurrence of post-CP hydrocephalus. Last, in our study, we defined ultra-early CP as occurring within 30 days post-DC, whereas conventional CP was defined as occurring after 30 days. The term conventional is meant to encompass both early (1–3 months) and late (>3 months) CPs.

### Statistical analysis

IBM SPSS software (version 26, Build 1.0. 0.1275; IBM Corp., Armonk, NY) was used for the entirety of our data analysis. Descriptive statistics were performed to demonstrate means for our non-parametric values, whereas Student's *t*-test was conducted to demonstrate the means for our continuous normal variables. Fisher's exact test was used to calculate the mean differences in numerical data between values of the ultra-early versus conventional CP groups. The *p* value required to achieve statistical significance in our study was set at *p* < 0.05.

### Ethical considerations

This study was reviewed and approved by the Denver Health Medical Center Institutional Review Board. Patient data collected from the electronic medical records were maintained in compliance with the Health Insurance Portability and Accountability Act of 1996 (HIPAA) guidelines. This study did not involve direct contact with patients, and therefore a waiver of informed consent was granted for the duration of our study.

## Results

A total of 124 CPs were performed at our institution. Seventy-seven patients met inclusion criteria for this study. The number of patients in both the ultra-early and conventional groups were similar (*n* = 39 and *n* = 38, respectively). Most patients were male (*n* = 58, 73.4%), and the mean age of patients in both the ultra-early CP and conventional groups were 42.90 ± 16.04 years and 35.90 ± 14.52 years, respectively. The primary indication for DC in both the ultra-early and conventional CP groups was TBI (ultra-early, 97.4%; conventional, 84.2%); other indications for DC included intracerebral hemorrhage, meningioma, and acute ischemic stroke. Table 1 depicts the demographics of all patients included in this study.

**Table 1.** Demographic Data Collected on the Patients, as Well as the Indications for Craniectomy among Our Patient Population

	Ultra-early group (*n* = 39)	Standard group (*n* = 38)
Mean age (years)	42.9	35.9
Sex (M/F)	32 M/7 F	26 M/12 F
Indication for craniectomy		
Trauma (%)	97.4 (*n* = 38)	84.2 (*n* = 32)
Non-trauma (%)		
Intracerebral hemorrhage	2.6 (*n* = 1)	7.9 (*n* = 3)
Meningioma	—	5.3 (*n* = 2)
Acute ischemic stroke	—	2.6 (*n* = 1)

The average GCS at the time of arrival to the ER was 7.28 ± 3.90 and 6.92 ± 4.14 for the ultra-early and conventional groups, respectively. The average time to CP in the ultra-early and conventional group were 17.70 ± 7.75 and 95.70 ± 65.60 days, respectively. Operative time was shorter in the ultra-early cohort compared to the conventional cohort (ultra-early, 2.40 ± 0.71 h; conventional, 3.00 ± 1.63 h; *p* = 0.0336). The incidence of post-CP hydrocephalus was also lower in the ultra-early cohort (ultra-early, 10.3%; conventional, 31.6%; *p* = 0.026). In the ultra-early cohort, 12.8% (*n* = 5) of patients required a return to the operating room (OR), whereas 28.9% (*n* = 11) of patients required a return to the OR in the conventional cohort. Indications for a return to the OR included surgical site infection, bone flap resorption, subdural fluid collection, and exposed hardware (Table 2). Overall rates of a return to the OR (ultra-early, 12.8%; conventional, 28.9%; *p* = 0.098), post-operative wound infection (ultra-early, 12.8%; conventional 13.2%; *p* = 0.99), and bone flap removal after the initial CP (ultra-early, 12.8%; conventional 21.1%; *p* = 0.33) were lower in the ultra-early cohort, though these data were not statistically significant (Table 2).

**Table 2.** Comparison in Outcomes between the Ultra-Early and Standard Cranioplasty Cohorts

	Ultra-early group (*n* = 39)	Standard Group (*n* = 38)	*p* value
Mean GCS upon ER arrival	7.28 ± 3.90	6.92 ± 4.14	0.69
Mean time to cranioplasty (days)	17.70 ± 7.75	95.70 ± 65.60	—
Mean case length (hours)	2.40 ± 0.71	3.00 ± 1.63	0.0336
Rate of post-operative wound infection	12.8% (*n* = 5)	13.2% (*n* = 7)	0.99
Rate of post-cranioplasty hydrocephalus	10.3% (*n* = 4)	31.6% (*n* = 12)	0.026
Rate of return to the OR	12.8% (*n* = 5)	28.9% (*n* = 11)	0.098
Indications			
Surgical site infection	5	7	
Bone flap resorption	—	1	
Subdural fluid collection	—	2	
Exposed hardware	—	1	
Rate of bone flap removal	12.8% (*n* = 5)	21.1% (*n* = 8)	0.33

GCS, Glasgow Coma Scale; ER, emergency room; OR, operating room.

## Discussion

This study investigated the efficacy, safety, and comparability of ultra-early CP versus conventional CP regarding rates of post-operative complications at a level 1 trauma center. Reduction of operative time and incidence of hydrocephalus in the ultra-early CP cohort were statistically significant. However, GCS score at admission, differences in rates of post-operative infections, return to the OR, and bone flap removal were not statistically significant. One study previously investigated ultra-early CP and found that both early (<90 days) and ultra-early (<42 days) timing of CP were not associated with higher rates of post-operative complications when compared to intermediate (91–180 days) or late (>180 days) CP.^16^ However, we believe that our study is the first to explicitly define the timing of ultra-early CP as occurring within 30 days post-DC, as well as demonstrate an isolated comparison in effectiveness and outcomes between ultra-early and early CP.

Our definition of ultra-early cranioplasty (<30 days) differs from other definitions used throughout the literature. For example, in the study by Kim and colleagues, they used the term very early to define CP as occurring <30 days, early to define CP performed from 30 to 60 days, late to define CP performed from 60 to 90 days, and more late to define CP performed after 90 days.^9^ Alternatively, Iaccarino and colleagues reported on the international consensus regarding the definitions of different CP timings. Ultra-early CP, as agreed upon, was defined as occurring up to 6 weeks after DC.^3^ This difference in definition of ultra-early CP as defined by Iaccarino and colleagues, which is more commonly used, as opposed to that of Kim and colleagues and our definition, should be noted given that such differences in terminology and definitions can vary by institution.

Regardless of the explicit definitions, the efficacy of various timings of CP has been extensively studied. Currently, no consensus exists as to when the most optimal time is to perform CP post-DC. The conventional wisdom is to allow complete healing at the incision site and resolution of cerebral edema before performing a CP to reduce the risk of wound infection and delayed hydrocephalus.^5,8^ However, there has been increasing evidence to support that earlier CP may be safe and effective at reducing both. Oh and colleagues conducted a retrospective analysis to investigate whether early CP (<90 days) increases rates of post-operative infection compared to late CP (>90 days). Their study found that infection rates with early CP were lower than with late CP (early, 7%; late, 20%; *p* = 0.02).^17^ A similar trend was reported by Bjornson and colleagues and Quah and colleagues regarding the reduction in rates of post-operative infection in early (<3 months and <12 weeks, respectively) versus late (>3 months and >12 weeks, respectively) CP (8% vs. 13%; *p* > 0.99 and 0% vs. 6%; *p* = 0.55, respectively), though these results were not statistically significant.^5,23^ Morton and colleagues found that CP performed in the very-early time frame (15–30 days) may minimize infection risks, whereas CP performed in the ultra-early time frame (<14 days) had a significantly increased risk of infection.^32^ Our study showed that patients who underwent ultra-early CP had lower rates of post-operative infections in comparison to the conventional cohort. Despite our data not achieving statistical significance in terms of showing reduced rates of infection in the ultra-early cohort, we believe our study demonstrates that ultra-early timing of CP is non-inferior to early CP. Whether or not there are key contributing factors that increase the risk for infection post-CP within 14 days remains to be elucidated by future studies.

A systematic literature review performed by Tasiou and colleagues reported evidence from several studies that early CP can also help to avoid post-operative hydrocephalus and the syndrome of trephined.^33^ These effects are thought to be mediated by restoration of the skull defect post-DC, given that this normalizes regional hemodynamic and metabolic derangements caused by the brain's exposure to elevated atmospheric pressures.^6^^,34^ Our study has shown that ultra-early CP significantly reduces the rate of post-operative hydrocephalus compared to early CP. Eaton and colleagues similarly reported a reduction in rates of post-operative hydrocephalus in their ultra-early subgroup (<42 days), as well as reduction in all other complications (seizure, hematoma formation, infection, or other) when compared to early (<90 days) or intermediate (91–180 days) groups.^16^ However, these findings did not achieve statistical significance. Contrary to our findings, CP within 90 days has been previously shown to increase the risk of hydrocephalus,^1,25^ and therefore it remains difficult to draw a definitive conclusion on the effects of earlier CP on hydrocephalus.

Our study also shows that ultra-early CP can significantly reduce operative times compared to early CP. Several studies report reductions in operative time with early CP when compared to late CP.^2,12,25^ Such findings have been attributed to having less scar tissue formation in earlier CP, allowing for easier establishment of the dissection plane of the scalp flap while replacing the autologous bone piece.^2,25^ Similarly, it is our observation that ultra-early CP allows for easier dissection, and therefore reduced operative times, as a result of less scar tissue formation.

This study is not without its limitations. Given the retrospective nature of our study, the level of evidence is much lower than that of a prospective study, and so it is difficult to determine temporality and causation regarding outcomes. This study was also performed at a single level 1 trauma center and, therefore, may suffer from a lack of external validity. Our retrospective study did not include an analysis of post-operative neurological function between the ultra-early CP and early CP cohorts. Findings of improved neurological outcomes with ultra-early CP would further strengthen the evidence for ultra-early CP as a safe and efficacious option post-DC. Patients who underwent ultra-early timed CP may have also had less severe injuries at initial presentation, which could have been a potential confounding factor in our analysis. As such, future studies should use a prospective study design or be conducted as a randomized controlled trial to further delineate the effects of ultra-early CP on post-operative outcomes.

## Conclusion

According to our results, ultra-early CP demonstrates a statistically significant reduction in the rate of post-operative hydrocephalus, as well as operative time, in comparison to early CP. However, ultra-early CP does not significantly reduce rates of post-operative infection, return to the OR, and bone flap removal. The timing of CP is a heavily debated neurosurgical topic. It is our hope that the results of this study add to the ongoing discussion regarding which timing would be most optimal to perform a CP. In the absence of high-quality prospective studies and randomized controlled trials, the timing of CP post-DC should remain a patient-centered consideration.

## Statement of Ethics

This study received Institutional Review Board (IRB) approval.

## Data Availability Statement

The data mentioned here are property of the Denver Health Medical Center and were accessed with the EPIC medical records system. All data generated or analyzed during this study are included in this article and/or its supplementary material files. Further enquiries can be directed to the corresponding author.

## Abbreviations Used

CP: cranioplasty
DC: decompressive craniectomy
ER: emergency room
GCS: Glasgow Coma Scale
OR: operating room
TBI: traumatic brain injury
